# Immunomodulatory Factors in Primary Endometrial Cell Cultures Isolated from Cancer and Noncancerous Human Tissue–Focus on RAGE and IDO1

**DOI:** 10.3390/cells10051013

**Published:** 2021-04-25

**Authors:** Joanna Tkaczuk-Włach, Witold Kędzierski, Ilona Jonik, Ilona Sadok, Agata Filip, Marta Kankofer, Wojciech Polkowski, Piotr Ziółkowski, Andrzej Gamian, Magdalena Staniszewska

**Affiliations:** 1 Diagnostic Techniques Unit, Collegium Maximum, Medical University of Lublin, Staszica 4/6, 20-081 Lublin, Poland; joannatwlach@gmail.com; 2 Department of Biochemistry, Faculty of Veterinary Medicine, University of Life Sciences in Lublin, Akademicka 12, 20-033 Lublin, Poland; witold.kedzierski@up.lublin.pl (W.K.); ilona.jonik@kul.lublin.pl (I.J.); marta.kankofer@up.lublin.pl (M.K.); 3 Laboratory of Separation and Spectroscopic Method Applications, Centre for Interdisciplinary Research, The John Paul II Catholic University of Lublin, Konstantynow 1J, 20-708 Lublin, Poland; ilona.sadok@kul.pl; 4 Department of Cancer Genetics with Cytogenetic Laboratory, Medical University of Lublin, Radziwillowska 11, 20-080 Lublin, Poland; agata.filip@umlub.pl; 5 Department of Surgical Oncology, Medical University of Lublin, Radziwillowska 13, 20-080 Lublin, Poland; wojciech.polkowski@uml.edu.pl; 6 Department of Pathomorphology, Wroclaw Medical University, Marcinkowskiego 1, 50-368 Wroclaw, Poland; piotr.ziolkowski@umed.wroc.pl; 7 Hirszfeld Institute of Immunology and Experimental Therapy, Polish Academy of Sciences, Weigla 12, 53-114 Wroclaw, Poland; andrzej.gamian@hirszfeld.pl; 8 SDS Optic S.A., Centrum ECOTECH-COMPLEX, Block A, 20-612 Lublin, Poland

**Keywords:** endometrium, primary cell culture, kynurenine, indoleamine 2,3-dioxygenase 1, RAGE

## Abstract

Background: Immune modulatory factors like indoleamine 2,3-dioxygenase 1 (IDO1) generating kynurenine (Kyn) and receptor for advanced glycation end-products (RAGE) contribute to endometrial and cancer microenvironment. Using adequate experimental models is needed to learn about the significance of these molecular factors in endometrial biology. In this paper we study IDO1 activity and RAGE expression in the in vitro cultured primary human endometrial cells derived from cancerous and noncancerous tissue. Methods: The generated primary cell cultures from cancer and noncancerous endometrial tissues were characterized using immunofluorescence and Western Blot for expression of endometrial and cancer markers. IDO1 activity was studied by Kyn quantification with High Performance Liquid Chromatography with Diode Array Detector. Results: The primary cultures of endometrial cells were obtained with 80% success rate and no major genetic aberrations. The cells retained in vitro expression of markers (mucin MUC1 and HER2) or immunomodulatory factors (RAGE and IDO1). Increased Kyn secretion was associated with cancer endometrial cell culture in contrast to the control one. Conclusions: Primary endometrial cells express immune modulatory factors RAGE and IDO1 in vitro associated with cancer phenotype of endometrium.

## 1. Introduction

Endometrial microenvironment constitutes very dynamic composition of several cell types, i.e., fibroblasts, epithelial, endothelial, and immune cells like macrophages or NK cells [1]. The architecture of the endometrium depends on the phase of menstrual cycle, status of pregnancy, or pathology [2] regulated by multiple factors, including hormones, immune suppression molecules, or residing microbiota [3,4,5]. Our knowledge on the significance of different components in endometrial pathology, including benign and malignant abnormalities, is still far from complete and awaits further studies using adequate models to mimic appropriate endometrial phenotype. Learning about the expression and relationship of important factors modulating immune status in human endometrium brings novel findings and help in the development of effective therapies.

The microenvironment of the endometrium is rich in mucins expressed on the surface of epithelial cells. These transmembrane glycoproteins, characteristic for endometrium, protect from pathogens, providing a barrier against environmental challenges. The expression patterns of different types of mucins have been extensively studied in human endometrium in order to verify their role in pathologies like infertility, endometriosis, and cancer [6,7,8]. MUC1 was associated with the increased proliferation [9] and metastatic potential [10] of cancer cells. Knowledge about changes in mucin expression patterns between normal and pathological states is still incomplete and needs further investigation [8].

The role of HER2 in different cancers has been well established [11]. It is a heterodimeric receptor activated upon binding with several ligands resulting in MAPK, PI3K, and PKC pathways activation [12]. HER2 overexpression is linked to poor prognosis in endometrial serous carcinoma patients [13]. Reports indicate that HER2 is an important cellular factor worth investigating as a therapeutic target for endometrial cancer [14,15]. Advances in targeted cancer therapy and successful treatments using anti-HER2 antibodies, i.e., trastuzumab, bring more interest in this potent cellular target also in the context of endometrium [16].

The receptor for advanced glycation end-products (RAGE) and indoleamine 2,3-dioxygenase 1 (IDO1) have been shown to contribute to regulation of immune status of endometrium are currently considered among new molecules of clinical relevance [17,18].

Soluble factors secreted by immune cells, with a prominent role of INF-γ, enhance IDO1 expression [19]. Activation of the kynurenine pathway (KP), responsible for the generation of several toxic metabolites called kynurenines, modulates local immune tolerance [20,21]. IDO1 activity is of great interest for scientists studying biology and pathology of endometrium [22]. Recently, KP has been also recognized as a novel factor in endometrial cancer tissue [23]. IDO expression and KP activation might be prognostic factors correlating with negative clinical outcome [24].

Contribution of surface receptor RAGE signaling is an additional component contributing to endometrial microenvironment [25,26,27]. This is a transmembrane receptor expressed on several cell types, including epithelial, endothelial, and immune cells activating migration, proliferation, and inflammatory response [26,28,29,30,31]. Several ligands activate RAGE, i.e., advanced glycation end-products (AGEs) or high-mobility group box 1 (HMGB1) and contribute to cancer progression [32,33,34]. Interestingly, RAGE is also present on endometrial cells and is upregulated in some endometrial cancer cells in vivo as well as in culture [27]. In that paper, knockdown of the receptor present on the endometrial cancer cells xenografted into mice resulted in decreased proliferation and vessel formation in vivo. Metabolic abnormalities in the case of insulin resistance (IR) and obesity have been shown to enhance negative effects of RAGE-mediated inflammation and oxidative stress [35]. Finally, RAGE is considered as a therapeutic target for the development of novel targeted drugs [17].

Identification and verification of the applicability of therapeutic targets in endometrial cancer require using adequate research models that in the case of human endometrium are scarce. Some examples of the xenotransplant models of human endometrial cells into mice have been described [36], but they present multiple challenges. The simpler models of the in vitro cultured cells derived from endometrium are adopted for this purpose [37]. Most of them are monocultures of one cell type that do not reflect the holistically complicated microenvironment of endometrium. Growing a monoculture of one cell type is also not trivial due to slow proliferation, thus modifications of the approach are continuously being explored. The most widely used endometrial cell lines are Ishikawa, HEC-1A, KLE (adenocarcinoma cells) and T-HESC (immortalized stromal cells) available from European Collection of Authenticated Cell Cultures (ECACC) and American Tissue Culture Collection (ATCC). Good alternatives for human are animal cells, like bovine monocultures of epithelial endometrial cells [38]. For the obvious reason, this is not an ideal model for studying human endometrial biology. Recently explored there are 3D human cell culture models called organoid culture [39,40] that might be also a promising set up for studying endometrial abnormalities or testing drugs. Some models contain several cell types to generate an interactive model supporting communication between different cell types. However, this model has some limitations due to the great heterogeneity of individual organoids that present different growth progress and require sophisticated microscopic techniques to be evaluated. In addition, using a medium without serum might favor expanding only some cell fraction and does not necessarily reflect in vivo tumor composition.

The aim of our study was to identify the possibility of studying the relevance of immune modulating factors within endometrial microenvironment in the patient-derived primary endometrial cells cultured in vitro. We focus especially on the selected aspects that contribute to endometrial cancer biology, such as IDO1 activity (expressed as l-Kyn level) and RAGE implicated with inflammatory and metabolic status. Expression of the clinically relevant cancer markers such as HER2 and MUC1 was also assessed in the context of the presented model of endometrial cancer. Our data, especially on the enhanced activity of kynurenine pathway, bring new insights into molecular events in endometrial cancer.

## 2. Materials and Methods

### 2.1. Patient Characteristic

A total of 22 tissue samples (Table 1) were obtained from women patients age 30–86 years, recommended to the surgical intervention due to a variety of endometrial abnormalities (benign and malignant), who were admitted to the Chair and Department of Gynecology and Gynecological Endocrinology, Medical University of Lublin, Poland. The used methodologies conformed to the standards set by the Declaration of Helsinki and the protocol was approved by the Bioethical Committee of the Medical University of Lublin. Written informed consent was obtained from each patient prior to the tissue collection. The study included patients referred for diagnostic curettage of the uterine cavity due to abnormal uterine bleeding with suspected endometrial cancer. Previous medical history indicated that some patients suffered from diabetes or hyperglycemia (Table 1).

After curettage of the uterine cavity, the tissue material subjected to immunohistochemistry was fixed in formalin, apart from a small piece further processed for in vitro cell culturing. Based on the histopathological results, patients were divided into a cancer group (10 patients) with endometrial adenocarcinoma type I and noncancerous group (12 patients) with non-neoplastic changes in the endometrium, i.e., straight hypertrophy of the endometrium, chronic endometrial inflammation, different degree of dysplasia, and endometrial polyp. One sample within the noncancerous group was from the benign change of endometrium (polyps) but there was metaplasia in the cervix identified at the same time (E9-17). There was also a case with mixed type endometrial changes, i.e., cancer cells and dysplasia (E4-11). In all patients with diagnosis of endometrial glandular cancer, attention was drawn to additional tests, such as fasting hyperglycemia, obesity-related history, menstrual irregularities (polycystic ovarian syndrome in the procreative age), and insulin resistance. In this respect, all studied patients showed classic risk factors for the development of endometrial cancer. Patients within the noncancerous group (without negative histopathology result in endometrial cancer) showed no history of the menstrual disorder, insulin resistance, and diabetes, with the exception of one woman with postmenopausal diabetes (Table 1, biopsy E1-6).

### 2.2. Immunohistochemistry

In addition to the diagnostic histopathology performed at the clinic, a portion of some specimens collected for the study was subjected to the immunohistochemistry (IHC) staining to confirm the status of the selected markers, i.e., CK8/18, HER2, RAGE, and IDO1 in the obtained specimen. The piece of the collected tissue was fixed in 4% formaldehyde in PBS (Phosphate-Buffered Saline) at the time of surgical removal and was embedded in paraffin (FFPE) for cutting into 4 μm sections. The slices mounted on poly-l-lysine-coated glass slides were first deparaffinized by heating at 60 °C and then immersed in xylene for 9 min, followed by antigen retrieval in DAKO Retrieval Solution pH 9.0 by heating in microwave Daewoo at 350 Watt and leaving at room temperature. The sections were next immunostained utilizing the ABC DAKO kit (Glostrup, Denmark). The endogenous peroxidase was first blocked with the Peroxidase-Blocking Solution (DAKO, Glostrup, Denmark) for 10 min at room temperature and washed twice with distilled water. After 15 min of blocking with Protein Block Serum-Free (DAKO, Glostrup, Denmark) at room temperature and washing with distilled water, the sections were incubated with one of the following antibodies: Cytokeratin 8/18 (CK8/18, clone EP1, DAKO, Glostrup, Denmark, ready to use), HER2 (DAKO, Glostrup, Denmark, Hercept Test), IDO1 (Merck, Darmstadt, Germany, diluted 1:80), RAGE (Merck, Darmstadt, Germany diluted 1:80) over night at 4 °C. The sections washed with PBS were next subjected to the 30 min incubation with DAKO, Glostrup, Denmark, EnVision + System-HRP at room temperature and after another PBS washing (2 × 5 min) the reaction was developed with 3,3′-diaminobenzidine tetrahydrochloride (DAB, DAKO, Glostrup, Denmark,) for 5 min. Finally, the slides were counterstained with hematoxylin-eosin (HE) and mounted under coverslips. The slides were observed under the Olympus BX51 microscope and pictures were recorded with the camera. The positive or negative reactivity was determined for each marker. IHC results of the collected endometrial tissue samples are presented in Table 2.

### 2.3. Isolation and Culture of Cells from Human Endometrium

The tissue subjected for cell culture immediately after removal was placed into a sterile container filled with Dulbecco’s Modified Eagle Medium/Nutrient Mixture F12 (DMEM/F12), supplemented with penicillin/streptomycin (pen/strep, Sigma, Saint Louis, MO, USA) and amphotericin (Sigma, Saint Louis, MO, USA), and was transported on ice for in vitro cell culturing. The tissue (pea size) was next placed on a plastic dish and cut into 1 mm pieces. The minced sample was collected with 5–10 mL of PBS containing pen/strep and amphotericin and transferred into a 15 mL conical tube for centrifugation at 200× *g* for 5 min. The resulting pellet was reconstituted in 2 mL of digestion solution containing 1 mg/mL of Collagenase I (Sigma, Saint Louis, MO, USA) in DMEM/F12 and incubated for 2–2.5 h at 37 °C to dissociate cells. The reaction was stopped by the addition of the complete culturing medium and cells were spun down at 200× *g* for 5 min. The cell pellet reconstituted in 0.5 mL of culturing medium was plated on the 6-well plate. When needed, sample of the endometrial tissue was treated with erythrocyte lysis buffer ELB (8.3 g ammonium chloride, 20 mg EDTA, 1 g sodium bicarbonate in 1 L) for 5–10 min at 37 °C. Finally, the cell pellet after centrifugation at 200× *g* for 5 min was reconstituted in 0.5 mL/well of culturing medium consisting of DMEM/F12 (PAN Biotech, Germany), 5% fetal bovine serum (FBS), 1 mg/mL bovine serum albumin (BSA), 10 ng/mL cholera toxin, 0.5 μg/mL hydrocortisone, 5 μg/mL insulin, 5 ng/mL epidermal growth factor (EGF), 5 μg/mL apo-transferrin (all from Sigma, Saint Louis, MO, USA), without antibiotics. The prepared culturing medium was used within 4 weeks. The plated tissue was incubated at 37 °C with constant 5% CO_2_ and routinely observed under the light microscope. The outgrown cells were passaged when reached 70% confluency and tested at passage 1–8.

The human ovarian cancer cell line SK-OV-3 from American Type Culture Collection (ATCC) was cultured at 37 °C with 5% CO_2_ in DMEM containing 4.5 g/L of d-glucose, supplemented with 10% (*v*/*v*) fetal bovine serum (FBS), 2 mmol/L l-glutamine and 1 U penicillin/streptomycin.

### 2.4. Determination of Population Doubling Time (PDT)

The cells were plated on 6-well plates at density 50,000 cells/well on individual plates that were subjected to cell counting on the particular day 1–10. The cells were collected from 3 wells by trypsinization for each time point and were counted manually. The Population Doubling Time (PDT) was calculated from the time frame of the cell linear growth using Equation (1):

(1) $$\mathrm{PDT}=\frac{T\times \ln2}{\ln\left(\frac{\mathrm{Xe}}{\mathrm{Xb}}\right)}$$

where T—hours between beginning and end point; Xe—number of cells at end time; Xb—number of cells at the beginning time.

### 2.5. Karyotyping

The cells were subjected to hypotonic shock by 20 min incubation with 0.075 mol/L KCl (Sigma, Saint Louis, MO, USA) at 37 °C. Then, cells were fixed with a cold mixture of methanol/acetic acid (3:1, *v*/*v*), sprayed on the microscope slides and left to dry on air. The RHG (R-bands by heating using Giemsa reagent) banding method, according to Sehested, has been employed for chromosome staining. Briefly, the slides were incubated with 1 mol/L PBS for 3–10 min at 88 °C and washed with ultrapure water. The Giemsa (AQUA-MED, Łódź, Poland) solution (5%) was applied to obtain R band staining. The GTG pattern (G-bands by trypsin using Giemsa was obtained by incubation with 0.25% trypsin solution in PBS (BIOMED, Lublin, Poland) for 2–3 min. After washing with PBS, the slides were incubated with 5% Giemsa. The chromosome pattern was analyzed under Nikon ECLIPSE-Ni light microscope. Data collected from 15–25 slides for each cell line were analyzed using Applied Spectral Imaging software.

### 2.6. Immunofluorescent Staining

The cells were cultured on chamber slides and after washing with PBS were fixed for 15 min at room temperature with 4% paraformaldehyde diluted in PBS. After 3 washing cycles with PBS, cells were permeabilized with 0.5% Triton X-100 for 15 min at room temperature and the chamber slides were incubated with a blocking solution (3% non-fat dry milk (NFDM), 1% BSA, 0.1% Triton X-100 in PBS) for 30 min at room temperature. Next, primary antibody against RAGE (Merck, Darmstadt, Germany, diluted 1:100), HER2 (Abcam, Cambridge, UK, diluted 1:200), Cytokeratin 8 (CK8, Biorbyt, Cambridge, UK, diluted 1:1000), vimentin (Abcam, Cambridge, UK, diluted 1:100), CD45-FITC clone HI30 (BD Pharmigen, Sa Jose, CA, USA, diluted 1:100), p53 (Abcam, Cambridge, UK, diluted 1:50), IDO1 (Merck, Darmstadt, Germany, diluted 1:200) or MUC1 (Dako, Glostrup, Denmark, diluted 1:100) were applied in the blocking solution, and chamber slides were incubated overnight at 4 °C. After washing (3 times) with PBS, the chamber slides were incubated for 1 h, at room temperature, with secondary Ab anti-rabbit IgG-AF555 or anti-mouse IgG-AF488 (ThermoFisher, Rockfold, IL, USA) for rabbit or mouse primary Ab, respectively. The washed 3 times with PBS chamber slides were next mounted with the mounting medium containing DAPI for nuclear staining. The slides were observed under Zeiss Axio Observer, LSM700 confocal microscope with 20× magnification or under the Nikon Eclipse Ti-2U Fluorescent microscope. The CK8- and vimentin-positive cells were counted from 3 fields as the % of total DAPI-stained nuclei.

### 2.7. Western Blotting

The cells were lysed with RIPA buffer containing protease inhibitor cocktail (Sigma, Saint Louis, MO, USA) and 100 nmol/L PMSF (Sigma, Saint Louis, MO, USA) followed by sonication. The lysate was loaded on 12% polyacrylamide gel and resolved in the presence of SDS. After protein transfer onto PVDF, the membrane was blocked with 5% NFDM/PBS containing 0.5% Tween-20 (PBS-T) for 1 h, at room temperature. The membrane was further left overnight at 4 °C with a mixture of rabbit anti-HER2 Ab (Abcam, Cambridge, UK 1: 500) and rabbit anti-β-actin Ab (Elabscience, Huston, TX, USA, 1:1000) diluted in 5% NFDM/PBS-T. Following 3 washing cycles with PBS-T, the membrane was incubated with a mixture of secondary Ab, i.e., anti-mouse IgG-AP and anti-rabbit IgG-AP, to detect mouse and rabbit Ab, respectively. The reaction was developed by incubation in a standard Alkaline Phosphatase substrate solution (Pierce Biotechnology, Rockford, IL, USA).

### 2.8. Quantification of l-Kyn Generated by Endometrial Cells Cultured In Vitro

l-Kyn secreted by endometrial cells was determined in conditioned medium from cultured in vitro cells as previously described with a modification [41]. Briefly, for analysis, an aliquot of conditioned medium (123.75 µL) was supplemented with 1.25 µL of 1 mg/mL stock solution of an internal standard (3-nitro-l-tyrosine, 3NT) and subjected to protein precipitation with 15 µL of 30% (*w*/*v*) trichloroacetic acid (Sigma, Saint Louis, MO, USA). After vortexing and centrifugation for 15 min at 14,000× *g*, 4 °C, the collected supernatant was gently evaporated to dryness in the Genevac EZ-2 Elite Personal Evaporator (Genevac Ltd., UK). The residual material was reconstituted in 25 µL an aqueous solution of 10 mmol/L ammonium acetate (pH 4.0) and transferred to chromatographic insert vial for immediate analysis on HPLC-DAD.

The analysis was carried on an Agilent Technologies 1200 Series high-performance liquid chromatograph equipped with autosampler (G1329A), quaternary pump (G1311A), vacuum degasser (G1322A), column thermostat (G1316A), and DAD (diode array detector, G1315D), equipped with an Agilent ChemStation software v.B.04.02. The chromatographic separation was accomplished on a Zorbax Eclipse Plus-C18 Rapid resolution HT (4.6 × 150 mm, 3.5 µm) analytical column coupled with a Zorbax Eclipse Plus-C18 (2.1 × 12.5 mm, 5 µm) Narrow Bore Guard Column (Agilent Technologies, New Castle, DE, USA) with temperature set at 40 °C. The elution was performed using solvent A (10 mmol/L ammonium acetate (Merck, Darmstadt, Germany) in water acidified to pH 4.0 with acetic acid (Merck, Darmstadt, Germany)) and solvent B (100% methanol (Merck, Darmstadt, Germany)) applying the following gradient program: 0–17 min—0% solvent B; 17–20 min—0–5% solvent B; 20–30 min—5–7% solvent B; 30–32 min—7–10% solvent B; 32–35 min—10–15% solvent B; 35–40 min—15–30% solvent B; 40–45 min—30–70% solvent B; 45–50 min—70–0% solvent B, and 50–55 min—0% solvent B. The flow rate was maintained at 0.5 mL/min and injection volume was 5 µL. The l-Kyn signal was monitored at wavelength 360 nm (retention time 15.86 min) and 3NT at 286 nm (retention time 28.26 min).

The calibration curve prepared in DMEM/12 was used to determine l-Kyn concentration. Matrix-matched calibration solutions (prepared analogously as experimental culture medium samples) contained fixed amounts of 3NT and variable concentrations of l-Kyn. Standards of crystalline l-Kyn and 3NT were obtained from Sigma-Aldrich (Saint Louis, MO, USA). Stock solutions of l-Kyn (1 mg/mL) and 3NT (1 mg/mL) were prepared daily by dissolving appropriate amounts of the substances in dimethyl sulfoxide (DMSO, Merck, Germany) or PBS (pH 7.7), respectively. A working solution of l-Kyn was prepared in DMSO. The blank response (l-Kyn in DMEM/F12) was subtracted from each sample to calculate l-Kyn concentration in the medium collected from cells. The limit of detection (LOD) and quantification (LOQ) estimated for l-Kyn in DMEM/F12 matrix were 0.074 and 0.24 nmol/mL, respectively.

### 2.9. Statistical Analysis

The Mann–Whitney U test was used to evaluate difference in concentration of l-Kyn present in culture medium of the in vitro grown cells isolated from cancer and the noncancerous endometrium. For calculations, the results above LOQ were considered. Data were processed using the PQStat software. Differences were considered significant at *p* < 0.05.

## 3. Results

### 3.1. Development of the Experimental In Vitro Model

We have successfully developed and secured for further studies several cultures of human endometrial cells obtained from patients with cancer (10 cultures) and noncancerous endometrium (12 cultures). The success rate of cell culture was 90% and 69% in the cancer and the noncancerous group, respectively.

**Figure 1:**
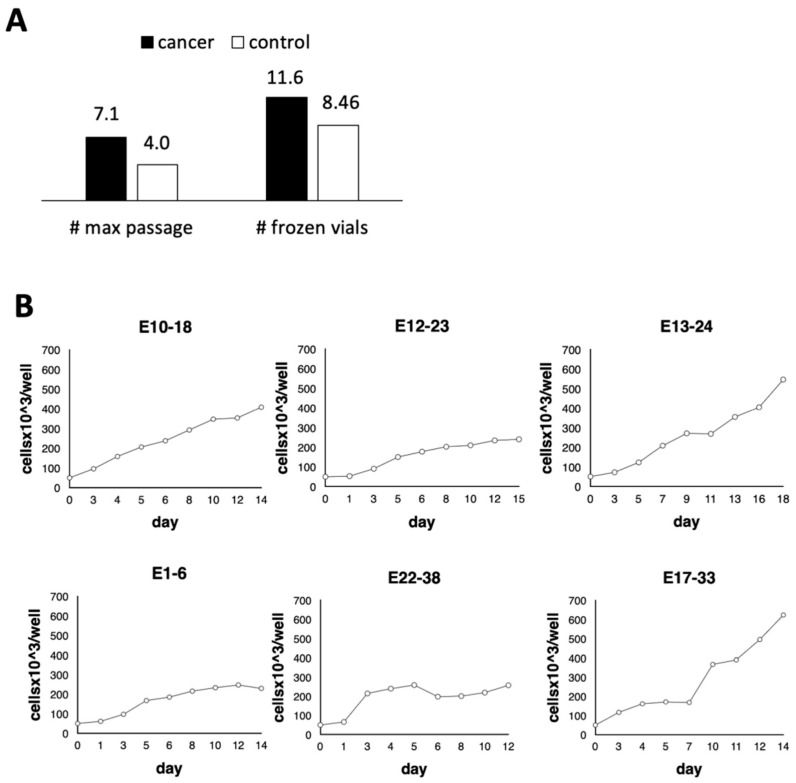
Potential of cell expansion in the in vitro culture conditions. (**A**) Comparison of the maximal number of passages and obtained number of frozen vials derived from cancer and the noncancerous endometrial tissue. (**B**) Growth kinetics of the selected endometrial cell cultures (cancer cells: E10-18, E12-23, E13-24 and control cells: E1-6, E22-38, E17-33) was assessed by plating 50,000 cells per well on 6-well plates and recording at the indicated time post-plating the cell number in the individual wells after trypsinization.

The limited amount of tissue mass (often large sample is unavailable) was an important determinant of the employed method of cell isolation. We started the cultures from a pea-sized sample of endometrial tissue. Such a sample without separation naturally contains different cell types present in the endometrial environment. Such an approach allows for retaining a composition of factors and cellular interactions which is natural for the organ and unique for the individual patient. To avoid fibroblast overgrowth, the medium components have been optimized to favor the growth of the epithelial cells. The complete culture medium contained DMEM/F12 with lower (5%) concentration of FBS was supplemented with BSA, cholera toxin (growth stimulator of epithelioid cells), insulin, apo-transferrin, hydrocortisone, and EGF. Additionally, in the first step of cell isolation, the tissue samples were minced and collagenase-treated for better cell disintegration and outgrowth. The endometrial samples that contained a substantial amount of blood were treated with ELB to deplete residual erythrocytes. Noteworthy, in order to assure better tissue adherence to the plate surface, the amount of medium at the beginning of the culture was kept at minimum. This minimized lifting-up of the pieces of tissue while transferring the plate and daily examination under the microscope. The first cell outgrowth was usually noticed about 2–5 days post plating. It was observed that the cells obtained from cancer tissue were easier to expand and resulted in higher number of passages as well as number of frozen vials, in contrast to the noncancerous cells (Figure 1A). Five out of twenty-two biopsies initially attempted to culture (23%) were not able to further expand in vitro, and eventually ceased. The cultures were carried through several passages, some even reached 15 passages, although the kinetics of growth varied (Figure 1B) and calculated Population Doubling Time (PDT) was from 28 to 67 h, when tested at passage around p3–p7.

**Figure 2:**
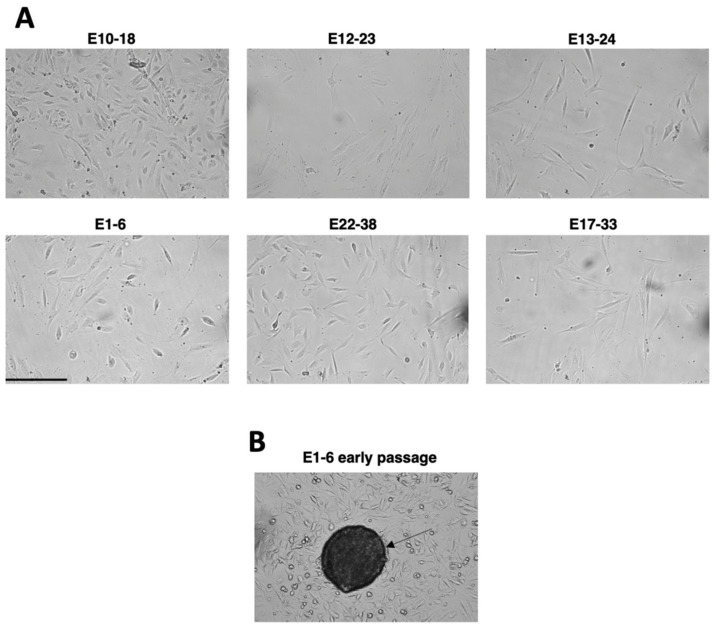
Morphology of the in vitro cultured endometrial cells. The cells derived from cancer (E10-18, E12-23, E13-24) and noncancerous (E1-6, E22-38, E17-33) tissue were cultured at p7 (**A**) and p0 (**B**); the arrow indicates 3D cellular aggregate; scale bar 50 µm.

The morphology of the growing in vitro cells was evaluated under the light microscope (Figure 2A). Appearance of the obtained cell cultures varied from typical epithelial morphology, cells with irregular shape, or mixture-containing cells with elongated morphology characteristic for fibroblasts. We have noticed that lowering serum concentration to 5% resulted in a less intense proliferation of fibroblast-like cells allowing for expanding other cell types present in endometrial tissue. Some of the isolates at the lower passages had a tendency to form the regular shape 3D structures similar to organoids reported elsewhere [39] that contained highly packed cellular mass (Figure 2B, arrow). These cellular aggregates were further dissociated during cell passaging and eventually the culture became a monolayer (Figure 2A, lower panel E1-6).

**Figure 3:**
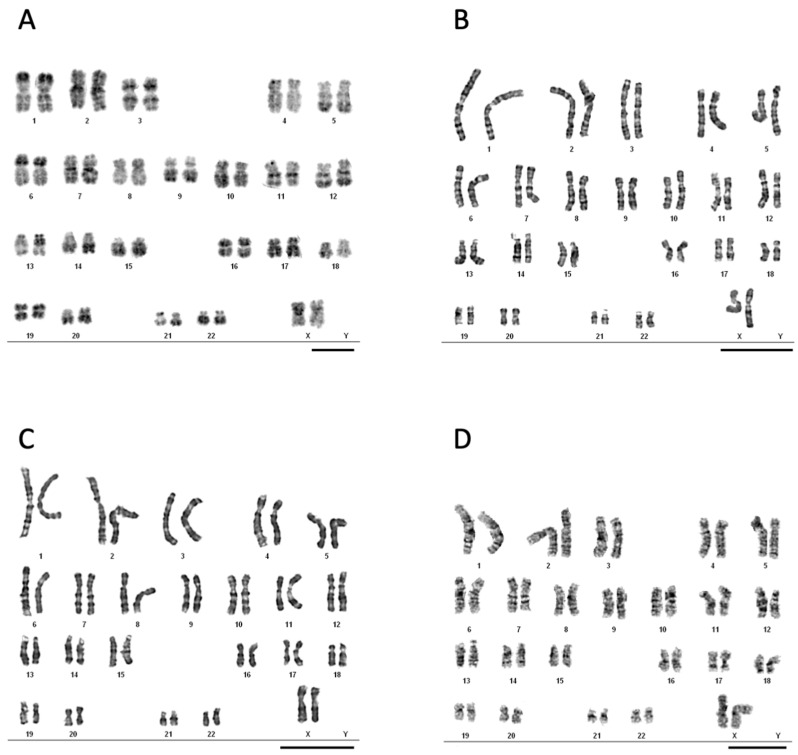
Karyotype characterization of cancer cells isolated from patients and cultured in vitro cells. The fixed on slide noncancerous endometrial cells (**A**) E1-6, (**B**) E22-38, and cancer endothelial cells (**C**) E10-18 and (**D**) E12-23 were stained for R-banding (RHG) and G-banding (GTG). The analysis was performed on 15–20 fields and showed a female pattern without abnormalities. The figure presents representative pictures for each cell culture. Scale bars 10 μm.

The karyotype of selected cultures isolated form noncancerous (E1-6, E22-38) and cancer (E10-18, E12-23) tissue have also been analyzed by G- and R-band staining [42]. In noncancer, as well as cancer isolates, we have reported normal number of chromosomes without aberrations as expected for women’s phenotype (Figure 3).

**Figure 4:**
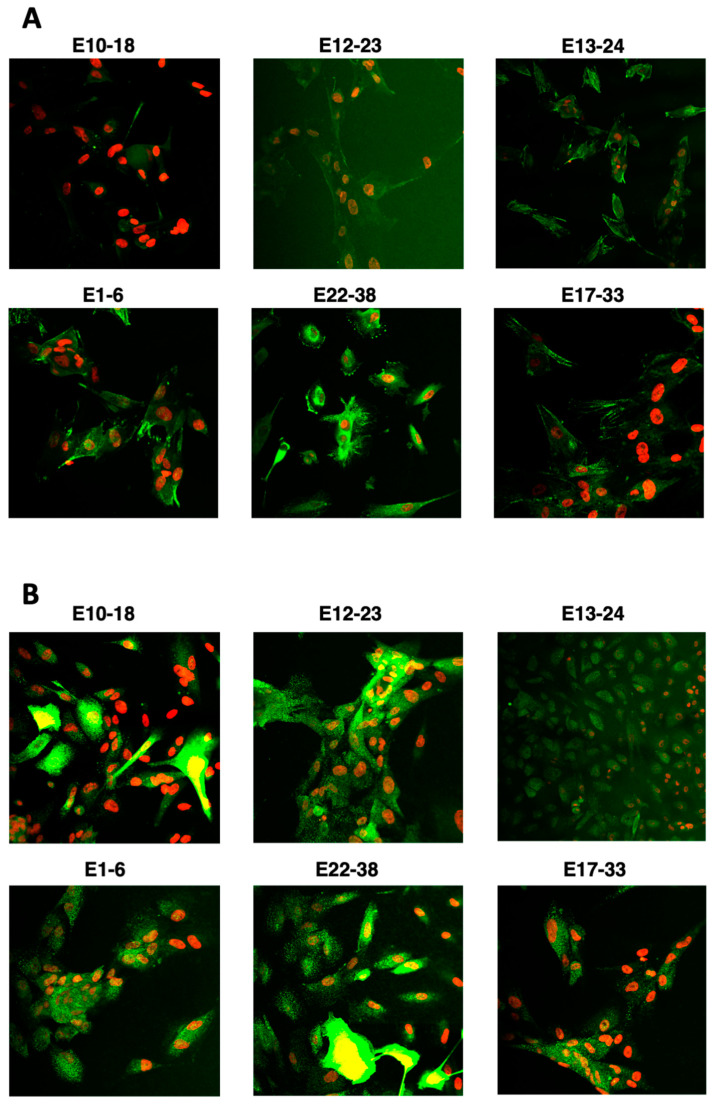
Expression of CK8 and MUC1 in the cultured in vitro endometrial cells. The cells derived from noncancerous (E1-6, E22-38, E17-33) and cancer (E10-18, E12-23, E13-24) tissue were cultured on chamber slides, fixed and immunostained with (**A**) anti-CK8 (green) or (**B**) anti-MUC1 Ab (green); nuclei were co-stained with DAPI (red).

To characterize the obtained cultures, we performed immunofluorescent staining for Cytokeratin 8 (CK8) that is abundantly present in epithelial cells and overexpressed on several types of cancers and in vitro cultured epithelial cells [43]. The cells cultured on chamber slides were fixed and stained with anti-CK8 antibody. Our results indicated that CK8 was expressed in most of the cells present in primary cultures isolated from cancer and noncancerous endometrial tissue (Figure 4A and Figure S1). It was with the agreement for the IHC staining performed on the corresponding tissue specimens preserved for this purpose (Table 2). All the tested tissue samples showed positive reactivity with anti-CK8/18 antibody.

Staining with anti-vimentin antibody (Figure S1) that indicates stroma cells showed 5–47.3% of positive cells depending on the culture. Some cells presented intense staining (Figure S1, arrow), while there were cells with weak or no vimentin expression (Figure S1, arrowheads and stars, respectively).

Finally, to assess contribution of immune cells, we used the anti-CD45 antibody to identify leukocytes (Figure S1). However, there were only few CD45+ cells present with a negligible number in cancer (E10-18, E12-23) and noncancerous cultures (E17-33).

We also observed a positive staining with anti-MUC1 antibody of the in vitro cultured endometrial cells, from the cancer and noncancerous group (Figure 4B). However, the expression of this sialoprotein was culture-dependent, with variable intensity and homogeneity of staining.

### 3.2. HER2 and RAGE Status of the Endometrial Cells Cultured In Vitro

**Figure 5:**
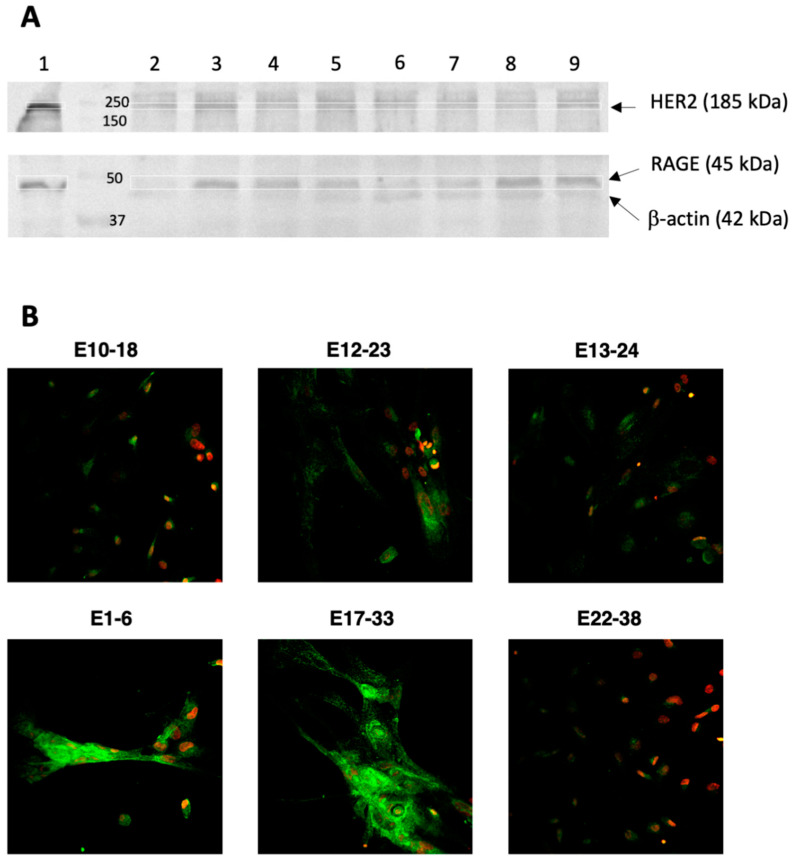
Expression of RAGE and HER2 on endometrial cells cultured in vitro. (**A**) WB was performed on cancer cell lysates (1) SK-OV-3, (2) E12-23, (3) E10-18, (4) E13-24, (5) E24-41, (7) E9-17, and noncancerous cells (6) E1-6, (8) E17-33, (9) E22-38 grown to 70–100% confluency, harvested in RIPA buffer, and subjected to WB analysis with anti-RAGE Ab and anti-β-actin Ab as a loading control. (**B**) The immunofluorescent staining of the cancer (E10-18, E12-23, E13-24) and noncancerous (E1-6, E17-33, E22-38) endometrial cells was performed with anti-RAGE Ab (green) and nuclei were co-stained with DAPI (red).

The expression of HER2 in the endometrial primary cell cultures was assessed by WB as the band of ~185 kDa. We have identified a relatively low amount of HER2 in endometrial cell cultures (Figure 5A, lane 2–9), as compared to the commercial cell line (SK-OV-3) of human ovarian cancer known for abundant expression of this receptor (Figure 5A, lane 1). In the primary cultures, there was variable expression of HER2 and mostly a rather weak band of HER2 was seen, even in the culture derived from HER2-positive biopsy (Figure 5, lane 2, E12-23). Moderate expression was observed in one culture (E10-18, lane 3) that was derived from HER2 negative biopsy. However, this specimen tested in IHC with the anti-HER2 antibody was negative (Table 2, sample 3). Importantly, there was no HER2 expression in cells derived from the control benign tissue (Figure 5A, lane 6, 8, 9). The specimen E22-38 (Figure 5A, lane 9) was also negative in IHC staining with anti-HER2 antibody (Table 2, lane 19).

Next, we aimed to determine the RAGE status of the obtained endometrial cell cultures. As demonstrated in Figure 5A, there was RAGE present in most tested lysates of the primary cultures (lane 3–9) and in the SK-OV-3 cells (lane 1). It was in agreement with the IHC staining for the corresponding tissues, i.e., E13-24, E24-41, E9-17 (Table 2, sample 5, 6, 15, respectively). There were some cell cultures (E10-18, E22-38) that showed RAGE expression in WB (Figure 5A, lane 3, 9) despite being derived from RAGE-negative tissue (Table 2, sample 3, 21). Interestingly, the lowest RAGE expression in WB was observed in the lysate of the cell culture derived from the HER2-positive biopsy (Figure 5A, lane 2).

RAGE expression in the primary cell cultures derived from the cancer and the noncancerous tissue was also observed by immunofluorescence after staining of the fixed cells with the same antibody that recognizes the extracellular RAGE protein domain (Figure 5B).

### 3.3. IDO1 Expression and l-Kynurenine Generation by the Primary Endometrial Cell Cultures

**Figure 6:**
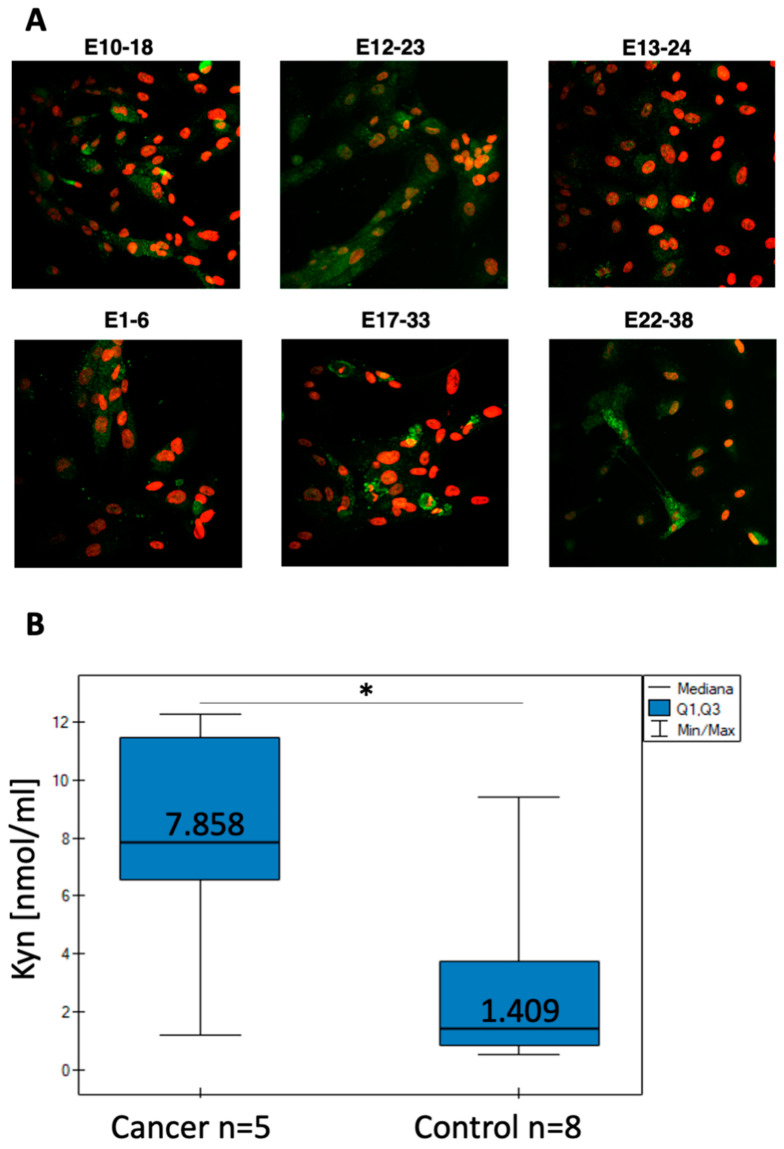
IDO expression and activity in endometrial cells cultured in vitro. (**A**) Immunofluorescent staining of cells obtained from cancer (E10-18, E12-23, E13-24) and noncancerous (E1-6, E17-33, E22-38) endometrial tissue probed with anti-IDO1 Ab (green) and co-stained with DAPI (red). (**B**) l-Kyn concentration in the medium from 5 cancer and 8 noncancerous (control) endometrial cell cultures was determined by HPLC-DAD. The Mann–Whitney U test was employed to compare median level of l-Kyn within cancer and noncancerous cells. Asterisk (*) indicates significant difference with *p* = 0.045.

The endometrial cells derived from cancer (Figure 6A, upper panel) and noncancerous (Figure 6A, lower panel) tissue were fixed on chamber slides and stained with anti-IDO1 antibody to observe protein expression. Most of the cells present in the tested cultures were found positive for IDO1 in IF, despite being derived from cancer or noncancerous tissue (Figure 6A). Interestingly, the positive IF result for cultured cells did not corelate with IHC staining of the original tissue. We observed no IDO1 staining of the original tissue available for IHC (Table 2, sample 4, 6, 21) although the corresponding derived cells displayed IDO1 expression (Figure 6A, E10-18, E13-24, E22-38). However, the enzymatic activity of the IDO1 enzyme measured indirectly as the level of l-Kyn production showed a difference between the cancer and noncancerous cells. Using the HPLC-DAD approach, we analyzed l-Kyn level in conditioned medium from 6 cancer and 9 noncancerous endometrial cultures (Table 2). Two cell samples, E21-37 and E14-25, contained l-Kyn below level of quantification (LOQ) of the applied HPLC-DAD method and were not included in statistical analysis. Comparison using the Mann–Whitney U test showed that cultures derived from cancer tissue secrete significantly greater amounts of l-Kyn (*p* < 0.05) than cells derived from the noncancerous endometrial tissue (Figure 6B). Higher l-Kyn secretion suggests more active IDO1 enzyme in the cancer-derived cells. It was also interesting that cells derived from the IDO1-negative biopsy secreted l-Kyn into the medium (E21-37, E11-21, E22-38), suggesting that the IHC staining of the tissue might not completely reflects the phenotype of cells that are present in tumor. This observation highlights the importance of analytical approach in determination of the IDO1 activity in cancer tissue.

## 4. Discussion

Immunotherapy presents a promise for more effective anti-cancer treatment [44], however, regulation of immune mechanism in cancer microenvironment is still not fully known. IDO1 is involved in immune regulation and in endometrial cancer it is often associated with the check point protein PD-L1 [18] as determined by IHC staining. In our study, IDO1 staining was observed only in some cancer tissues and in some noncancer ones as well, which is in agreement with the previous reports [18,45]. Interestingly, in the in vitro cultures, derived from the endometrial tissue, the enzyme was active regardless of the IHC-negative IDO1 status of the original tissue (Table 2), as quantified by measuring of l-Kyn secreted to culturing medium. We recorded l-Kyn concentration to be higher in medium from the cancer group compared to the noncancer derived cultures (Figure 6B) translating into the higher enzyme activity. We believe our paper is the first demonstration that in vitro cultured primary endometrial cells recapitulate IDO1 overexpression seen in tissue stained by IHC (Table 2, sample 3). Kynurenine secretion by cells derived from IHC-negative tissue for IDO1 suggests underestimation of IDO1 status in the original tissue. The primary cultures have the advantage over the established cancer cell lines and consist of a mixture of epithelial, stromal cells, and other residual cell types. A possible scenario is enhanced proliferation of the IDO1 expressing stromal cells [46] in vitro. Kynurenine might also be generated by other tryptophan degrading enzymes like IDO2 and TDO [19]. The origin of l-Kyn generation by the endometrial cell cultures remains to be verified. However, analytical approaches of IDO1 determination, i.e., the used in this study HPLC-DAD might better reflect the status of IDO1 enzyme in the tumor and should be considered in final tissue assessment.

The presented endometrial cell cultures as demonstrated in Figure 5 showed expression of RAGE. The signaling initiated by RAGE ligands like advanced glycation end-products (AGEs), that accumulate in hyperglycemia and metabolic abnormalities, mediate oxidative stress and induce inflammation [25] associated with endometriosis [47,48] and endometrial cancer [27]. RAGE-initiated signaling is also considered as an immune modulating factor in cancer microenvironment, thus we tested RAGE expression in the cultured in vitro human endometrial cells. There was a difference in original tissue RAGE expression compared to the corresponding cell cultures. Most cultures expressed RAGE (determined by WB) although some tissues (Table 2, sample 4 21) remained negative for RAGE in IHC. Interestingly, the lowest amount of RAGE was seen for the cell culture derived from the HER2-positive tissue (Figure 5A, lane 2). This RAGE-HER2 inverse correlation was also observed in commercial SK-BR-3 breast cancer cell line known to overexpress HER2, while in the other breast cancer cell line MDA-MB-231 with low HER2 expression there was high RAGE (data not shown). The same low RAGE expression was seen in the tissue of the HER2-negative breast cancer subtype [49].

The presented in Figure 5B SK-OV-3 cell line showed expression of both receptors HER2 and RAGE. This implies that RAGE expression might be promoted in the in vitro cultured endometrial cells, unless there is low HER2 expression. This might be different for other cancers, such as ovarian cancer, where RAGE and HER2 coexist at high level (SK-OV-3 cells).

It would be also worth investigating in the future whether the negative IHC staining for RAGE observed in the studied inhere tumor tissue biopsies is associated with an increased level of AGEs that upon binding to RAGE can hamper tissue staining with anti-RAGE antibody. On the other hand, since hyperglycemia induces RAGE expression through ROS generation [50], it might be possible that in cancer patients with co-existing hyperglycemia (like in the present studies) there is an upregulation of RAGE in cancer cells that is further sustained during in vitro cell culture.

In vitro culture of the established cell lines, usually derived from cancer cells or immortalized primary cells [51], is an approach providing a constant source of material with minimal variability. However, it has several disadvantages over the tissue directly obtained from patient, i.e., it is usually monoculture [37] and hard to completely mimic in in vivo conditions. While the established cell lines are relatively easy and predictable to work with, they might be used to study only some aspects of the tumor environment, i.e., they lack several cellular components and factors supplied by other cell types present in the original cancer tissue.

Here we present the data on the in vitro cultured primary cells that can be used as models for studying the immune components of the endometrial environment. Using the presented approach, we were able to propagate not only cancer but also noncancerous endometrial cells, although the cancer cells had a higher success rate and greater expansion properties compared to the cells isolated from noncancerous tissue (Figure 1). This was supported by the differential staining with anti-CK8 and vimentin markers indicating epithelial and stromal cells, respectively. In our cultures, the majority of cells showed epithelial phenotype, although some relevant number of stromal cells was also present (Figure S1, Table 3). Since we did not perform CK8/vimentin co-staining, it is difficult to say that the vimentin+ cells are CK8+; however, it might be assumed based on calculated percentage in both types of staining (Table 3). It can be explained by cytoskeletal rearrangement in the rather dynamically behaving partial EMT cells undergoing epithelial-to-mesenchymal transition (EMT) [52].

Importantly, we did not observe chromosomal changes in our primary cultured cells, indicating that the model system does not introduce major gene aberrations (Figure 3), as it is observed in the immortalized cell cultures [37,53]. The genetic changes occurring during cell immortalization might negatively influence the experimental results and are often raising a question of the specificity of the observed effects [54].

The previously described markers important in endometrial receptivity [55] and overexpressed in cancer metastasis [10], such as MUC1, we confirmed to be abundant also in the endometrial cells cultured in vitro in our hands (Figure 4). The model cells also show expression of HER2 protein in most of the cancer cultures but not in cells derived from benign tissue. Surprisingly, the cells derived from the cancer tissue diagnosed as HER2+ also showed a low level of HER2 staining (Figure 5B, line 2), suggesting that the number of cells overexpressing HER2 was not dominant in this isolate.

Furthermore, the generated number of cells was sufficient to characterize the obtained culture and perform multiple assays, including protein expression, analysis of factors involved in immune regulation (kynurenine), inflammatory pathways, and metabolic abnormalities (RAGE) (Figure 4, Figure 5 and Figure 6).

The cultured cells displayed heterogeneous and changing morphology allowing for the 3D structure formation that mimics the native endometrial microenvironment (Figure 2B). This is important for studying endometrial cancer differentiation processes since the changes in cellular architecture mark the potency to metastasis with the involvement of multiple factors, i.e., TGF-β in epithelial-to-mesenchymal transition (EMT). The opposite effect is implicated also in the regeneration of endometrium [56] or embryo implantation [57]. Thus, the presented in vitro model with no need for additional extracellular matrix components unlike other models [53] will be useful to study mechanisms governing immune response in the endometrial microenvironment.

Finally, in cells isolated from cancerous tissue, we have shown increased generation of l-Kyn (Figure 6B) that is the first stable metabolite of the immune regulatory pathway mediated by IDO1. The enzyme expression has also been confirmed in the cultured cells (Figure 6A), likewise expression of HER2, another cancer marker associated with 30% of endometrial cancers [58]. This is an important observation indicating that the cultured cells retain malignant phenotype with high IDO1 [24,44] and HER2 expression observed in vivo [59]. Together, it makes this model suitable for identification and testing novel immunotherapeutics for HER2- and IDO1-associated pathology shown to be important in endometrial cancer [45] or endometriosis [22].

## 5. Conclusions

The described model of primary human endometrial cells presents an opportunity for investigating biology of endometrium, especially the immune component (IDO1 and RAGE), in normal and cancer cells.

## Figures and Tables

**Table 1:** Characteristics of the study group.

No.	Biopsy	Group	Age	Diagnosis
1	E2-7	Cancer	72	Undifferentiated carcinoma G2, Type II; hyperglycemia
2	E4-11	Cancer	59	Adenocarcinoma, Type I, G-1, endometrial polyp, dysplasia; hyperglycemia
3	E9-17	Cancer	48	Type II, G-2, metaplasia in cervix; hyperglycemia
4	E10-18	Cancer	41	Adenocarcinoma, G-1 and hyperplasia without atypia; hyperglycemia
5	E12-23	Cancer	86	Type II, G-3 (Carcinoma papillare serosum, HER2+); hyperglycemia
6	E13-24	Cancer	44	Adenocarcinoma, Type I, G-2 with hyperplasia complex atypica; hyperglycemia
7	E24-41	Cancer	65	Adenocarcinoma, Type I, G-1; hyperglycemia
8	E21-37	Cancer	66	Adenocarcinoma, Type I, G-2; hyperglycemia
9	E16-30	Cancer	49	Adenocarcinoma, Type I, G-2; hyperglycemia
10	E20-35	Cancer	70	Adenocarcinoma, Type I, G-1; hyperglycemia
11	E1-6	Noncancerous	76	Endometrial polyp, chronic inflammation of the endometrium; postmenopausal diabetes
12	E3-8	Noncancerous	48	Chronic inflammation of the endometrium
13	E5-12	Noncancerous	30	Endometrial polyp, chronic inflammation of the endometrium
14	E6-14	Noncancerous	47	Endometrial polyp, Endometrial hyperplasia without atypia with chronic inflammation
15	E7-15	Noncancerous	39	Endometrial necrosis
16	E11-21	Noncancerous	58	Endometrial polyp; hyperglycemia
17	E14-25	Noncancerous	63	Endometrial hyperplasia without atypia
18	E17-33	Noncancerous	48	Endometrial hyperplasia without atypia
19	E18-34	Noncancerous	44	Endometrial hyperplasia without atypia
20	E19-36	Noncancerous	66	Endometrial polyp
21	E22-38	Noncancerous	43	Endometrial hyperplasia without atypia, chronic inflammation of the endometrium
22	E23-40	Noncancerous	39	Endometrial hyperplasia without atypia

**Table 2:** Immunochemistry (IHC) of the tissue and l-Kyn level generated by in vitro cultured cells determined by High-Performance Liquid Chromatography with Diode Array Detector (HPLC-DAD).

No.	Biopsy	Group	IHC Staining				l-Kyn [nmol/mL]
			CK8/18	HER2	RAGE	IDO1	
1	E2-7	Cancer	na	na	na	na	1.22
2	E4-11	Cancer	na	na	na	na	7.86
3	E9-17	Cancer	+	−	+	+	12.26
4	E10-18	Cancer	+	−	−	−	na
5	E12-23	Cancer	na	na	na	na	11.44
6	E13-24	Cancer	+	−	+	−	6.55
7	E24-41	Cancer	+	−	+	+	na
8	E21-37	Cancer	+	−	+	−	<LOQ
9	E16-30	Cancer	+	−	+	+	na
10	E20-35	Cancer	na	na	na	na	na
11	E1-6	Noncancerous	na	na	na	na	1.03
12	E3-8	Noncancerous	na	na	na	na	0.54
13	E5-12	Noncancerous	na	na	na	na	0.87
14	E6-14	Noncancerous	na	na	na	na	3.43
15	E7-15	Noncancerous	+	−	−	+	na
16	E11-21	Noncancerous	+	−	−	−	9.39
17	E14-25	Noncancerous	na	na	na	na	<LOQ
18	E17-33	Noncancerous	na	na	na	na	4.78
19	E18-34	Noncancerous	na	na	na	na	0.77
20	E19-36	Noncancerous	+	−	+	+	na
21	E22-38	Noncancerous	+	−	−	−	1.78
22	E23-40	Noncancerous	na	na	na	na	na

na—samples not analyzed; + indicates tissue with positive IHC reactivity; − indicates tissue with negative IHC reactivity; l-Kyn was quantified in the cell-culturing medium by HPLC-DAD; <LOQ—under limit of quantification of the applied HPLC-DAD method.

**Table 3:** Identified % of CK8+ and vimentin+ cells in cancer (E10-18, E12-23, E13-24) and noncancerous (E1-6, E17-33, E22-38) cells stained on chamber slides with anti-CK8 or anti-vimentin antibody and co-stained with DAPI.

Cell Culture	% CK8	% Vimentin
E10-18	99.3 ± 1.0	18.2 ± 7.4
E12-23	98.2 ± 3.4	5.3 ± 3.1
E13-24	98.3 ± 3.4	32.9 ± 7.3
E1-6	99.4 ± 1.0	11.1 ± 4.4
E17-33	93.8 ± 6.2	23.1 ± 20.7
E22-38	96.2 ± 3.2	47.3 ± 30.0

## Data Availability

Not applicable.

## Supplementary Materials

The following are available online at https://www.mdpi.com/article/10.3390/cells10051013/s1, Figure S1: Expression of CK8, vimentin, and CD45 on endometrial cells cultured in vitro.
