# Supplementary material for: Immunomodulatory Factors in Primary Endometrial Cell Cultures Isolated from Cancer and Noncancerous Human Tissue–Focus on RAGE and IDO1
Source: Cells. 2021 Apr 25;10(5):1013. doi: 10.3390/cells10051013 (PMC8145962; doi:10.3390/cells10051013)
Supplement: Supplementary file 1 [file cells-10-01013-s001.zip › cells-1162025-supplementary.pdf]

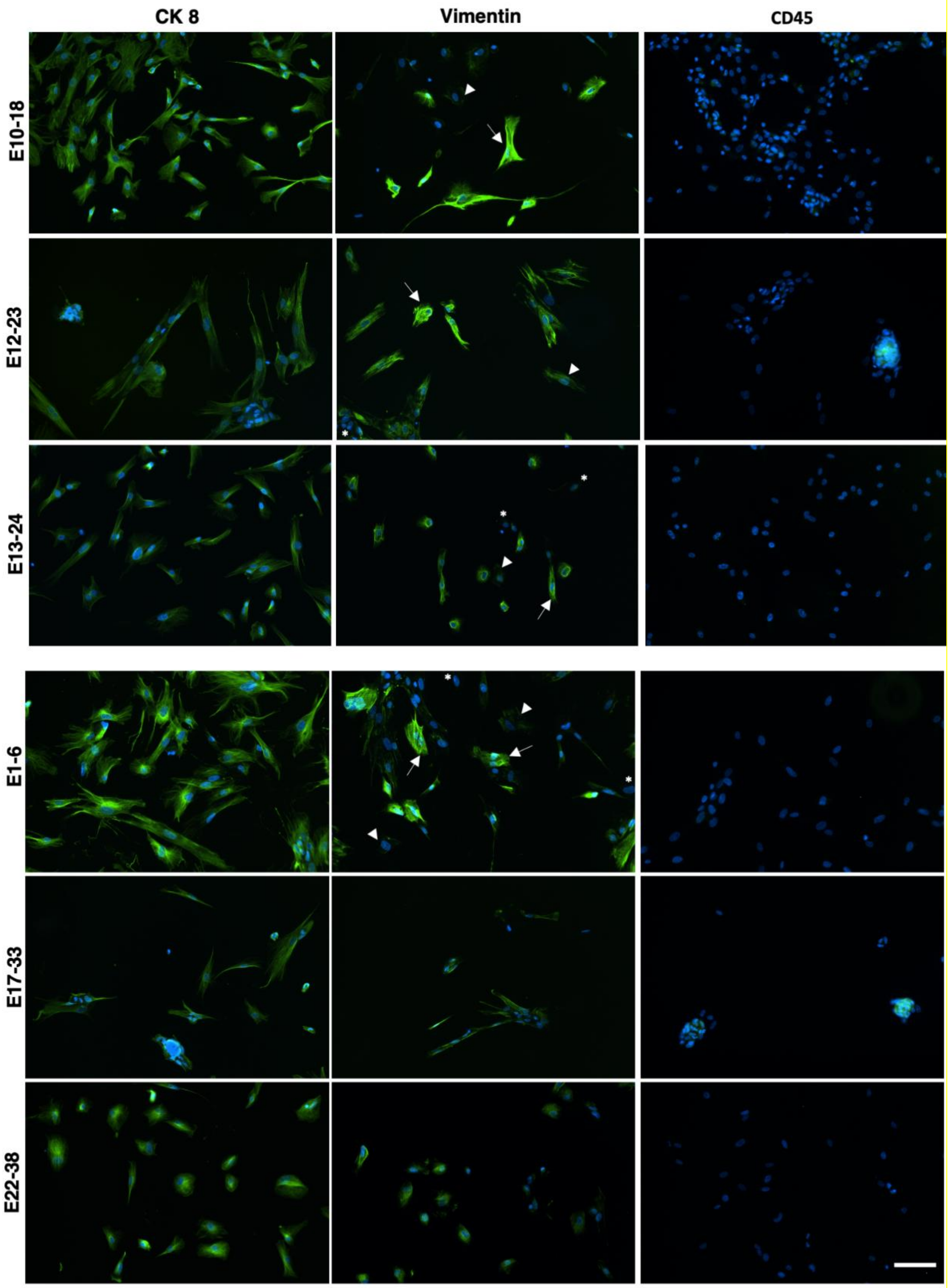

Figure S1. Expression of CK8, vimentin, and CD45 on endometrial cells cultured in vitro. Cells isolated from cancer (E10-18, E12-23, E13-24) and noncancerous (E1-6, E17-33, E22-38) endometrium were stained for CK8, vimentin and CD45 (green) and co-stained with DAPI (blue). Scale bar 100 μm; Arrow—intense positive, arrowhead—weak positive; star—negative staining for vimentin.
